# The evaluation of next‐generation sequencing assisted pathogenic detection in immunocompromised hosts with pulmonary infection: A retrospective study

**DOI:** 10.1111/crj.13542

**Published:** 2022-10-18

**Authors:** Donghui Zhang, Shujing Chen, Ying Wang, Dongni Hou, Cuicui Chen, Linlin Wang, Xinjun Tang, Xiaoyan Chen, Lin Tong, Yuye Zhang, Jinjun Jiang, Yuanlin Song

**Affiliations:** ^1^ Department of Pulmonary Medicine, Zhongshan Hospital Fudan University Shanghai China; ^2^ Shanghai Respiratory Research Institute Shanghai China; ^3^ Department of Critical Care Medicine, Zhongshan Hospital Fudan University Shanghai China; ^4^ Shanghai Institute of Infectious Disease and Biosecurity Shanghai China; ^5^ National Clinical Research Center for Aging and Medicine, Huashan Hospital Fudan University Shanghai China; ^6^ Shanghai Key Laboratory of Lung Inflammation and Injury Shanghai China; ^7^ Jinshan Hospital of Fudan University Shanghai China

**Keywords:** immunocompromised host, next generation sequencing, outcome, pulmonary infection

## Abstract

**Introduction:**

Pulmonary infections are frequent in immunocompromised hosts (ICH), and microbial detection is difficult. As a new method, next‐generation sequencing (NGS) may offer a solution.

**Objectives:**

This study aimed to assess the impact of NGS‐assisted pathogenic detection on the diagnosis, treatment, and outcomes of ICH complicated by pulmonary infection and radiographic evidence of bilateral diffuse lesions.

**Methods:**

This study enrolled 356 patients with ICH complicated by pulmonary infection that were admitted to Zhongshan Hospital, Fudan University, from November 17, 2017, to November 23, 2018, including 102 and 254 in the NGS and non‐NGS groups, respectively. Clinical characteristics, detection time, rough positive rate, effective positive rate, impact on anti‐infective treatment plan, 30‐day/60‐day mortality, and in‐hospital mortality were compared.

**Results:**

NGS‐assisted pathogenic detection reduced detection time (28.2 h [interquartile range (IQR) 25.9–29.83 h] vs. 50.50 h [IQR 47.90–90.91 h], *P* < 0.001), increased positive rate, rate of mixed infection detected, effective positive rate, and proportion of antibiotic treatment modification (45.28% vs. 89.22%, 4.72% vs. 51.96%, 21.65% vs. 64.71%, 16.54% vs. 46.08%, *P* < 0.001). The NGS group had a significantly lower 60‐day mortality rate (18.63% vs. 33.07%, *P* = 0.007). The difference in the Kaplan–Meier survival curve was significant (*P* = 0.029). After multivariate logistic regression, NGS‐assisted pathogenic detection remained a significant predictor of survival (OR 0.189, confidence interval [CI], 0.068–0.526).

**Conclusion:**

NGS‐assisted pathogenic detection may improve detection efficiency and is associated with better clinical outcomes in these patients.

## Graphical abstract

In this study, we discovered that next‐generation sequencing (NGS)‐assisted pathogenic detection may improve detection efficiency and is associated with better clinical outcomes in immunocompromised pulmonary‐infected patients with radiographic evidence of bilateral diffuse lesions.

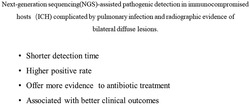

## 1 INTRODUCTION

The number of hospitalized patients considered immunocompromised hosts (ICH) continues to increase because of the prevalent use of chemotherapy for cancer, organ transplantation, glucocorticoids, and other immunosuppressive therapies for various disease conditions. For these patients, pulmonary infection is one of the most frequent and fatal complications.^1^ Respiratory infections and drug toxicity were major concerns in over two‐thirds of ICH cases, and mortality may reach 50–80% if accompanied by respiratory failure.^2^, ^3^ Traditional infectious and opportunistic pathogens account for a substantial proportion of infections in these patients. Danés et al.^4^ discovered opportunistic fungal pneumonia in 38% of non‐human immunodeficiency virus (HIV) ICH patients with etiologically confirmed pneumonia. Therefore, accurate and rapid microbial detection can improve outcomes in these patients. However, the current standard microbial culture has two major drawbacks: low sensitivity and the amount of time it takes to return results.^5^

Molecular diagnostic methods, such as polymerase chain reaction (PCR) and next‐generation sequencing (NGS), may provide a solution to this problem. Pathogen sequences can be sorted using deep sequencing and data mining without using a specific primer.^6^ Several studies have demonstrated the medical benefits of NGS. Guo et al.^7^ and Ai et al.^8^ used NGS to monitor disease progression and therapeutic effectiveness. Long et al.^9^ utilized NGS to detect bacteria in blood samples from patients with sepsis in the intensive care unit (ICU) and discovered greater sensitivity than culture. Pan et al.^10^ used a case series to reveal the effectiveness of NGS in detecting pathogens in bronchoalveolar lavage fluid (BALF) samples of ICH with community‐acquired pneumonia. Miao et al.^11^ analyzed 511 specimens (16 types of samples, BALF and sputum samples, approximately 60%, and blood samples, 7.4%) using NGS and traditional culture and discovered that NGS had higher sensitivity and was less likely to be affected by antibiotic exposure. However, obtaining good‐quality BALF or sputum samples remains difficult in many patients, especially in ICH with pulmonary infection; thus, blood specimens may be the main sample type. Therefore, developing a sensitive detection method utilizing NGS for various sample types would assist clinicians in the diagnosis and treatment of ICH with pulmonary infection; however, this has not been studied previously. Because NGS is more expensive than traditional diagnostic methods, it must be evaluated in an appropriate population, such as ICH, with subsequent clinical outcomes and detection efficiency. In this study, we aimed to assess the impact of NGS‐assisted pathogenic detection on the diagnosis, treatment, and outcomes of ICH with pulmonary infection and radiographic evidence of bilateral diffuse lesions.

## 2 MATERIALS AND METHODS

### 2.1 Patient population

This study was conducted at Zhongshan Hospital, Fudan University, a tertiary medical center in Shanghai. Clinical Research Ethics Committee of Zhongshan Hospital, Fudan University (Shanghai, China) approved this study (B2017‐122, B2018‐205). This study enrolled consecutive immunocompromised patients between November 17, 2017, and November 23, 2018. Inclusion criteria involved patients: (1) aged ≥16 years old, (2) with pulmonary infection, (3) who required routine standard microbial detection (either blood samples or respiratory tract specimens), and (4) whose pulmonary imaging revealed bilateral diffuse lesions. Immunocompromise was defined in accordance with the hospital‐acquired pneumonia or ventilator‐associated pneumonia (HAP/VAP) guidelines of the Japanese respiratory society 2009.^12^ Patients had been immunocompromised for at least 1 month before enrollment.

Anti‐infection treatment usually takes 24–48 h to take effect, and it is difficult to determine whether the treatment was effective if patients died before the microbial results or less than 48 h after the microbial results. Therefore, we excluded those who died before the microbial results were reported after 48 h. When comparing the sequence of microbial results and patient death, mycobacteria or negative Aspergillus culture results were not considered. In our hospital, at least 168 h is necessary for a negative Aspergillus culture result, and mycobacteria culture may take approximately 6 weeks. In this study, ICH was required for the timely adjustment of anti‐infection treatment. Some patients may die before these two types of results are obtained, and these results had little effect. Only one HIV‐positive patient was transferred to the Shanghai Public Health Clinical Center after the test, and we could not follow‐up on the outcome. Thus, this patient was excluded.

### 2.2 Study design and data collection

This was a retrospective study. During the research period, physicians screened patients who met the criteria and asked the research assistants to assess the patients as a group. After obtaining informed consent, patients received free NGS detection (limited by the detective capacity, only a portion of the patients who met the criteria received this extra detection). If the patient survived for at least 48 h after the microbial result, the patient was included in the NGS group. For the non‐NGS group, we screened medical records during the research period and selected all patients who met the study's criteria but did not receive NGS detection. Patient baseline data, including age, sex, acute physiology and chronic health evaluation II (APACHE II) score, type of immunosuppression, experimental examination, treatment, length of hospital stay, and survival data, were collected from medical records and electronic databases. In both groups, sputum, BALF, blood, lung tissue, throat swabs, and pleural effusion samples were collected before antibiotic use. In the non‐NGS group, only conventional microbiological methods, including smears and cultures of normal bacteria and fungi, were used. Subsequently, positive cultures were subjected to drug‐sensitivity tests. Samples from suspected cases of mycobacteria were also sent for acid‐fast bacillus testing and mycobacterial culture. Based on clinical features, serological tests and PCR were used in some cases. These tests included 1,3‐β‐D‐glucan, Cryptococcus capsular antigen, G‐lipopolysaccharide, antibodies against *Mycoplasma pneumoniae* and *Legionella pneumophila*, detection of nine main pathogens in the respiratory tract, CMV‐IgG, CMV‐IgM, CMV‐DNA, influenza A/B, and T‐SPOT. Empiric antibiotics were adjusted based on the results of the microbiological tests. For the NGS group, samples were sent to the BGISEQ‐500/100 NGS and conventional pathogenic detection platforms simultaneously, and empiric antibiotics were adjusted later. The follow‐up period was limited to 60 days. Data S1 contains the procedures for NGS detection.

### 2.3 Criteria for some follow‐up content

The detection time is the time duration from sample collection to result (Mycobacteria culture and negative Aspergillus culture not included). If there were more than one sample, we recorded the time of the first positive sample or the first sample (all samples were negative) for the non‐NGS group, and for the NGS group, we recorded the average time of all the samples sent for NGS detection.

An NGS report was considered positive if at least one pathogen was detected that met the following three criteria^11^, ^13^: (i) pathogenicity was detected in the lungs; (ii) at least two copy reads were detected; and (iii) if the pathogen was detected in both the negative control sample and the test specimen, the amount of nucleic acid in the test specimen should be at least five times higher.

Effective positive result is a result required overall consideration of NGS results, traditional microbial results, previous literature, other experiments or imagological examinations, and clinical characteristics by physicians. In this study, we considered an uncertain clinical microbial diagnosis as negative for ease of analysis.

Modification of antibiotic treatment (MAT): Antibiotic treatment was adjusted based on the results of microbial detection.

### 2.4 Statistical analysis

Data were analyzed using SPSS 24.0 (SPSS, Chicago, IL, USA). For continuous variables, we used a two‐tailed independent Student's *t*‐test or the Wilcoxon rank‐sum test. Non‐parametrically distributed variables were assessed using chi‐square (χ2) test or Fisher's exact test. The *R* and *C* χ2 test and Bonferroni method were used to compare the proportions of different ICH types between the two groups. Univariate and multivariate analyses were performed using logistic regression. The Kaplan–Meier survival analysis was used to compare survival rate between different groups. Statistical significance was set at *P* < 0.05.

## 3 RESULTS

### 3.1 Study participants and baseline characteristics

**FIGURE 1:**
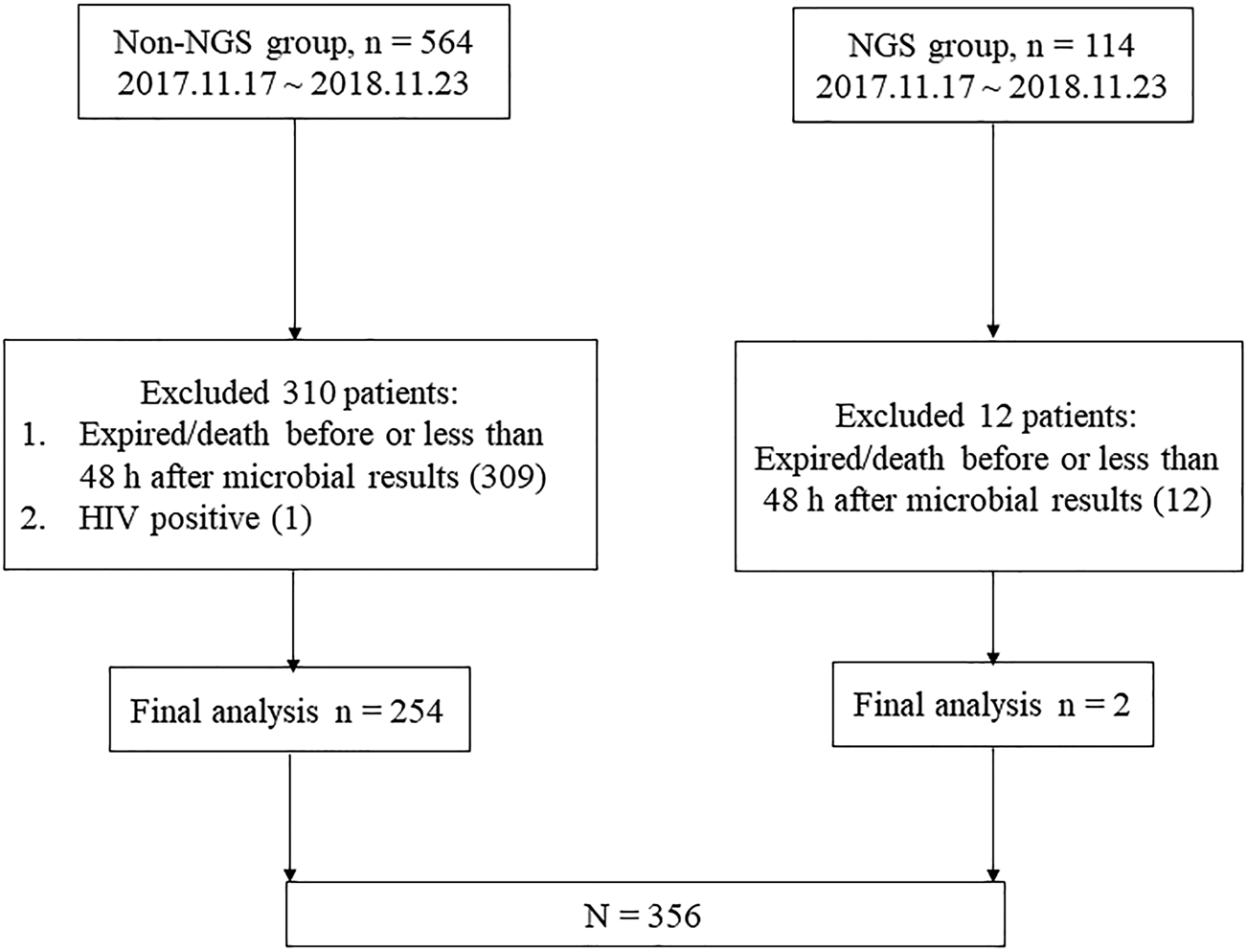
Patient selection. Abbreviations: NGS, next‐generation sequencing; HIV, human immunodeficiency virus

Six‐hundred seventy‐eight patients met the enrollment criteria, and after, 322 patients were excluded (321 patients died before or within 48 h after microbial results and one HIV‐positive patient), and 356 patients were included in the final analysis (Figure 1). No significant differences in age, sex, APACHE II scores, proportion of patients with severe pneumonia, multidrug‐resistant bacterial infection, or invasive mechanical ventilation were detected between the two groups. However, the type of immunocompromise differed significantly between the two groups (*P* < 0.001). After further comparison using the Bonferroni method, we discovered the cause of this difference. The proportion of hematological neoplasms was lower in the non‐NGS group (16.93% vs. 36.27%, *P* < 0.05), and the proportion of chemotherapy or radiotherapy for solid tumors was higher (16.93% vs. 3.92%, *P* < 0.05) (Table 1).

**TABLE 1:** Baseline characteristics

Characteristic	Non‐NGS group (*n* = 254)	NGS group (*n* = 102)	*P* value
Age^a^	63 (52.75–7.25)	58.50 (45.75–67.50)	0.055
Gender (Male %)	24.02% (61)	40.20% (41)	0.624
APACHEII^a^	10 (7–15)	10 (8–13)	0.695
HAP/VAP	11.02% (28)	6.86% (7)	0.233
Severe pneumonia	30.31% (77)	35.29% (36)	0.361
Invasive ventilation	15.75% (40)	16.67% (17)	0.831
Multi‐drug resistant	10.24% (26)	8.82% (9)	0.686
Type of immunocompromise			<0.001
DM (not compilation)	18.90% (48)	14.71% (15)	ns
Hematological neoplasms	16.93% (43)	36.27% (37)	*
Chronic kidney disease^b^	18.50% (47)	11.76% (12)	ns
Rheumatic disease	14.96% (38)	18.63% (19)	ns
Organ transplantation	7.09% (18)	3.92% (4)	ns
Chemotherapy or radiotherapy of solid tumor	16.93% (43)	3.92% (4)	*
Hypoproteinemia/hepatic failure	6.69% (17)	10.78% (11)	ns

Abbreviations: APACHE, acute physiology and chronic health evaluation; CI, confidence interval; HAP/VAP, hospital‐acquired pneumonia or ventilator‐associated pneumonia; DM, diabetes mellitus; NGS, next‐generation sequencing; ns, the difference was not significant (by Bonferroni method); OR, odds ratio.

^a^ Data are shown with median interquartile range (IQR).

^b^ Renal failure or chronic kidney disease that requires glucocorticoid or immunosuppressant.

* Significant difference (Bonferroni's method).

### 3.2 NGS performance

**FIGURE 2:**
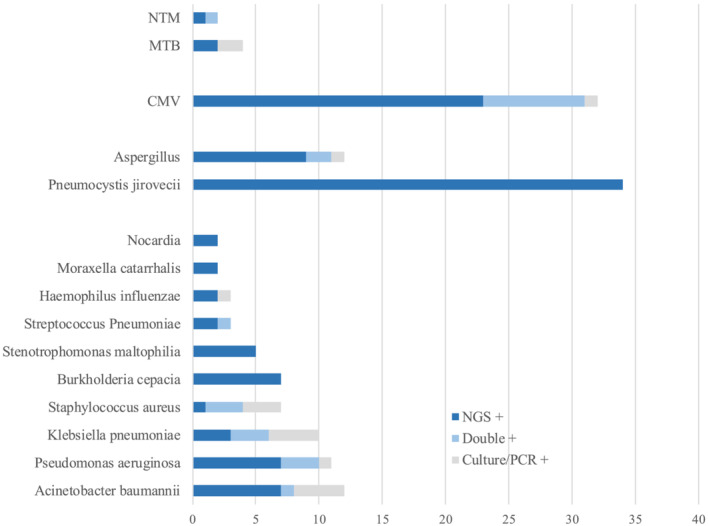
Important pathogens detected by NGS and traditional microbial method in the NGS group. Abbreviations: NTM, non‐tuberculous mycobacterial; MTB, *Mycobacterium tuberculosis* ; CMV, cytomegalovirus; NGS, next‐generation sequencing; PCR, polymerase chain reaction

The detection time was shorter in the NGS group than in the non‐NGS group (28.2 h IQR 25.9–29.83 h vs. 50.50 h IQR 47.90–90.91 h, *P* < 0.001). The positive rate, effective positive rate, rate of mixed infection detected, and proportion of modified antibiotic treatment were significantly higher in the NGS group (89.22% vs. 45.28%, 51.96% vs. 4.72%, 64.71% vs. 21.65%, 46.08% vs. 17.71%, *P* < 0.001) (Data S2). In total, 123 samples from the NGS group were sent for NGS detection, including 79 blood samples, 28 BALF samples, 15 sputum samples, and 1 pleural effusion sample. The positive rate was not significantly different among the three sample types (blood, sputum, and BALF), and NGS had a higher positive rate than microbial culture in each sample type (86.08% vs. 11.54% in blood samples, 89.29% vs. 26.92% in BALF samples, 100% vs. 54.93% in sputum samples, *P* < 0.001). In total, 31 types of bacteria (only those with reported evidence of pathogenicity in the lungs), 9 types of fungi, 9 types of viruses, and 2 types of mycobacteria were identified in the NGS group. Pathogens that are frequently detected include *Pneumocystis jeroveci* (PJ) and cytomegalovirus (CMV). In the non‐NGS group, culture‐positive *Candida albicans* was the most common pathogen, which was clinically insignificant (important pathogens detected in the NGS group are listed in Figure 2, and the complete catalog is in Data S3).

### 3.3 Concordance between results of NGS and traditional methods

In the NGS group, results of the NGS and traditional methods were compared. NGS results were positive in 89.25% of cases, with 46.24% of cases being double‐positive (NGS positive and culture positive). The negative predictive value was 81.82% ± 40.45%. Subsequently, we compared NGS and culture results for these double‐positive cases. Match, partly matched, and mismatched cases accounted for 21.51%, 7.53%, and 9.67% of all cases, respectively. Mismatch cases were mostly caused by culture‐positive *C. albicans*, and after removing this interference, the proportion declined to 2.15% (Data S4 and S5).

### 3.4 Outcomes of the two groups

**FIGURE 3:**
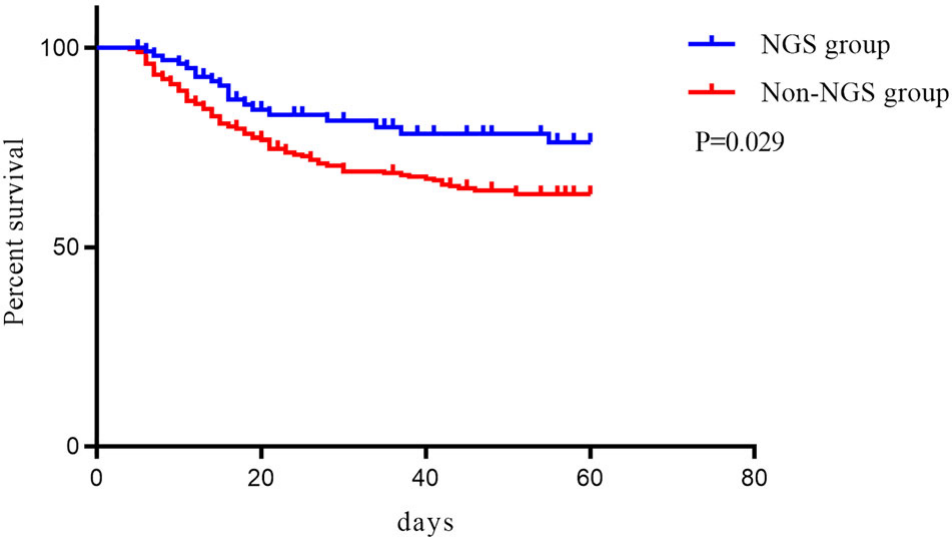
Survival curve of the NGS and non‐NGS groups

**FIGURE 4:**
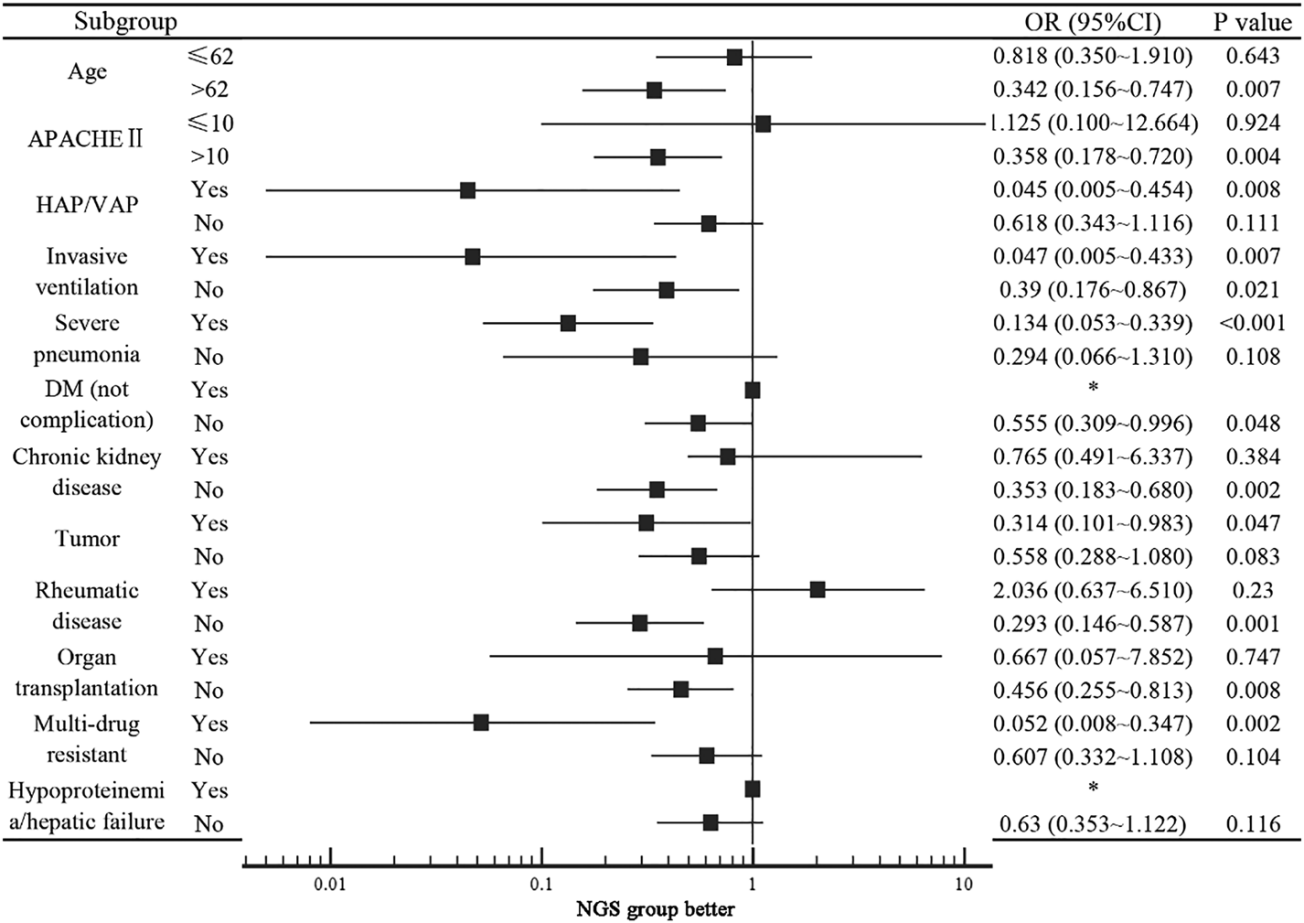
Subgroup analysis for 60‐day mortality. Abbreviations: APACHE, acute physiology and chronic health evaluation; CI, confidence interval; HAP/VAP, hospital‐acquired pneumonia or ventilator‐associated pneumonia; DM, diabetes mellitus; NGS, next‐generation sequencing; OR, odds ratio

There was a significant difference in 30‐/60‐day and in‐hospital mortality between the NGS and traditional detection groups (16.67% vs. 28.35%, 18.63% vs. 33.07%, 18.63% vs. 33.07%, *P* < 0.05). This difference was also significant in Kaplan–Meier curve (Figure 3). Multivariate logistic regression analysis (Table 2) indicated that HAP/VAP (OR 6.41, *P* = 0.038), invasive ventilation (OR 17.271, *P* < 0.001), and APACHE II scores (OR 1.899, *P* < 0.001) were risk factors for 60‐day mortality, and NGS detection was a predictor of survival (OR 0.189, confidence interval [CI], 0.068–0.526). Subsequently, subgroup analysis was used to identify the appropriate population that benefitted from this detection method in terms of survival (Figure 4). Potential survival benefits included age >62 years (OR 0.342, *P* = 0.007), APACHE II score >10 (OR 0.358, *P* = 0.004), severe pneumonia (OR 0.047, *P* = 0.007), radiotherapy or chemotherapy for malignant tumors (OR 0.314, *P* = 0.047), and multidrug resistant infection (OR 0.052, *P* = 0.002).

**TABLE 2:** Univariate and multivariate logistic regression analysis for 60‐day mortality

Variate	Univariate analysis			Multivariate analysis		
	OR	95% CI	*P* value	OR	95% CI	*P* value
Age	1.046	1.028–1.064	<0.001	1.01	0.98–1.036	0.61
Multidrug resistant	6.66	3.120–14.197	<0.001	3.13	1.62–8.4	0.568
HAP/VAP	5.774	2.748–12.130	<0.001	6.41	1.11–37.04	0.038
Invasive ventilation	33.154	14.244–77.166	<0.001	17.271	3.899–76.502	<0.001
Hematological neoplasms	0.319	0.161–0.632	0.001	0.701	0.211–2.335	0.563
Chemotherapy or radiotherapy of solid tumor	1.177	0.608–2.280	0.629	1.709	0.533–5.487	0.368
APACHE II score	1.909	1.659–2.197	<0.001	1.899	1.605–2.224	<0.001
Gender	1.081	0.674–1.716	0.746	1.179	0.515–2.702	0.697
NGS detection	0.463	0.264–0.813	0.007	0.189	0.068–0.526	0.001

Abbreviations: APACHE, acute physiology and chronic health evaluation; CI, confidence interval; HAP/VAP, hospital‐acquired pneumonia or ventilator‐associated pneumonia; NGS, next‐generation sequencing; OR, odds ratio.

## 4 DISCUSSION

In this study, we discovered that NGS‐assisted pathogenic detection improved diagnosis and treatment and was associated with better clinical outcomes in immunocompromised pulmonary‐infected patients with radiographic evidence of bilateral diffuse lesions. This method reduced microbial detection time, increased detection efficiency, provided more evidence for modifying antibiotic treatment, and was associated with decreased mortality. To our knowledge, this is the first study to evaluate the detection performance, and the impact on treatment and clinical outcomes of NGS‐assisted pathogenic detection in ICH with pulmonary infection. This is also the first study to explore the relationship between molecular detection in blood samples and clinical outcomes of ICH.

Timely and precise pathogenic diagnosis is essential in ICH. This aids the identification of opportunistic pathogens, and deescalates antibiotics over time.^14^, ^15^, ^16^ Previous studies have indicated that conventional culture examination may require 3–5 days for the final result, and NGS only requires 2–3 days.^3^, ^16^ We discovered similar results when comparing detection times. The sequencing procedure only takes approximately 24 h; hence, the total time to obtaining results by improving submission process can be shortened in the future.^17^, ^18^, ^19^ As expected, NGS produced a satisfactory positive rate compared to conventional methods. We used the concept of “effective positive rate” to determine the real pathogen, which required an overall consideration of NGS results, traditional microbial results, previous literature, other experimental or imageological examination, and clinical characteristics by physicians. Based on this concept, the NGS group had a higher rate of detected mixed infection and proportion of modified antibiotic treatment.

The advantage of NGS was further demonstrated by comparing the detection performance of the three main types of samples. Miao et al.^11^ discovered no difference in the positive rate between the three types of samples, which may give clinicians more options. However, only a few blood samples were included, and only the non‐tuberculous mycobacterial‐positive rate was compared between groups.^11^ In many cases, obtaining ICH sputum samples is difficult, leaving blood samples as the only viable option for diagnostic testing. CMV and PJ, which are often unidentified using traditional microbial culture methods, were prevalent in ICH using NGS. This is consistent with the results of a study by Pan et al.^10^ In addition, Aspergillus, which was identified by NGS in approximately 10% of all samples, was unidentified using the traditional method. These results suggest the need to consider all possible pathogens in ICH. The negative predictive value was relatively high (81.82% ± 40.45%) was similar to the negative predictive value for NGS (89.2% ± 7.6%) in a study by Parize et al.^18^ study that compared traditional culture and NGS in the microbial diagnosis of all types of ICH infections using NGS.

Shorter microbial detection times, higher detection efficiency, and timely and reasonable antibiotic treatment adjustments may explain the better outcomes of the NGS group. Several studies have demonstrated the positive impact of rapid diagnosis and antibiotic management in bloodstream infection cases.^19^, ^20^, ^21^ Recently, Xie et al.^22^ discovered that NGS pathogenic detection reduced mortality in ICU patients with severe pneumonia. This was a retrospective study with a relatively small sample size, and the detection time was not reported. Here, we attempt to confirm and explain this conclusion in a study using a larger sample size. We focused on ICH, in whom the infection diagnosis is more difficult using conventional diagnostic methods.

After the multivariate logistic regression analysis, NGS remained a significant predictor of survival. In the subgroup analysis to determine the appropriate population for NGS detection, patients over 62 years of age or with high APACHE II scores received a survival benefit. Similar to the study by Xie et al.,^22^ this detection method was associated with reduced mortality in patients with severe pneumonia in our study. Our study was underpowered to draw conclusions specific to patients with multidrug resistant infections.

Our findings suggest that NGS‐assisted pathogenic detection works well in immunocompromised pulmonary infection patients with radiographic evidence of bilateral diffuse lesions, and that patients over 62 years or with high APACHE II scores (≥10) may benefit more. However, this study had several limitations. First, it was a non‐randomized clinical trial single‐center study, population size was not very large and patients were not enrolled prospectively. Second, not all patients in the NGS group underwent NGS detection for blood samples. Third, NGS detection in this study only covered DNA, thus eliminating potential viruses. This study provides strong evidence to justify further studies, including randomized trials, to determine whether NGS can improve clinical decision‐making and subsequent outcomes in ICH with pulmonary infection.

## CONFLICT OF INTEREST

The authors have no conflict of interest to declare.

## ETHICS STATEMENT

This study was approved by the Clinical Research Ethics Committee of Zhongshan Hospital, Fudan University (Shanghai, China) (B2017‐122, B2018‐205) and informed consent was obtained from every participant. Informed consent was obtained from individual or guardian participants.

## AUTHOR CONTRIBUTIONS

DH Z, SJ C, JJ J, and YL S designed the study and the idea of this article; SJ C, DN H, DH Z, and JJ J enrolled patients; CC C, LL W, XJ T, XY C, L T, and YY Z collected data; DH Z, SJ C, and Y W conducted statistics and analysis of results; DH Z and Y W also conducted manuscript writing and editing; SJ C, JJ J, and YL S revised and corrected the manuscript.

## Supporting information


**Data S1.** Supporting Information

**Data S2.** Supporting Information

**Data S3.** Supporting Information

**Data S4.** Supporting Information

**Data S5.** Supporting Information

## Data Availability

All data generated or analyzed during this study are included in this published article (and its supplementary information files).
