# Supplementary material for: The evaluation of next‐generation sequencing assisted pathogenic detection in immunocompromised hosts with pulmonary infection: A retrospective study
Source: Clin Respir J. 2022 Oct 18;16(12):793–801. doi: 10.1111/crj.13542 (PMC9716706; doi:10.1111/crj.13542)
Supplement: Supplementary file 1 — Data S1. Supporting Information [file CRJ-16-793-s001.docx]

Supplementary material 1-Procedures for NGS detection

Blood samples, sputum samples and BALF samples were stored at 4℃, lung tissue samples were stored at [liquid](javascript:;) [nitrogen](javascript:;). All of the samples were sent to BGISEQ-500/100 NGS platform with in 4h. The volume of blood samples were 3~5ml per case，for sputum the volume was 0.5~3ml，and for BALF the volume was 5~10ml. After put at indoor temperature for 3~5min, the blood samples were centrifuged 10min (1600g, 4℃). Then the plasma was transferred into sterile tubes for next procedure. The sputum samples were digested by 0.1%DTT for 30min at indoor temperature before extraction of DNA. Tissue samples were mixed with 0.5g glass beads(0.5mm) and shook intensively for 2 minutes before next procedure. There was no such pretreatment for BALF samples. Before DNA extraction, 0.5ml sample fluid and 1g glass bead were mixed in a 1.5ml centrifuge tube and be stirred for 30min. DNA was extracted by TIANamp Micro DNA Kit (DP316, TIANGEN BIOTECH, Beijing, China).

The sequence was amplified by PCR before analysis. After exclusion of the sequence less than 35bp and host sequence (identified by Burrows-Wheeler match), the rest was matched with the reference database. The reference was download from NCBI which included 4189 kinds of virus, 2358 kinds of bacteria, 199 kinds of fungus and 135 kinds of parasite that may cause infection in human being.
