# Supplementary figures and images for: The evaluation of next‐generation sequencing assisted pathogenic detection in immunocompromised hosts with pulmonary infection: A retrospective study
Source: Clin Respir J. 2022 Oct 18;16(12):793–801. doi: 10.1111/crj.13542 (PMC9716706; doi:10.1111/crj.13542)

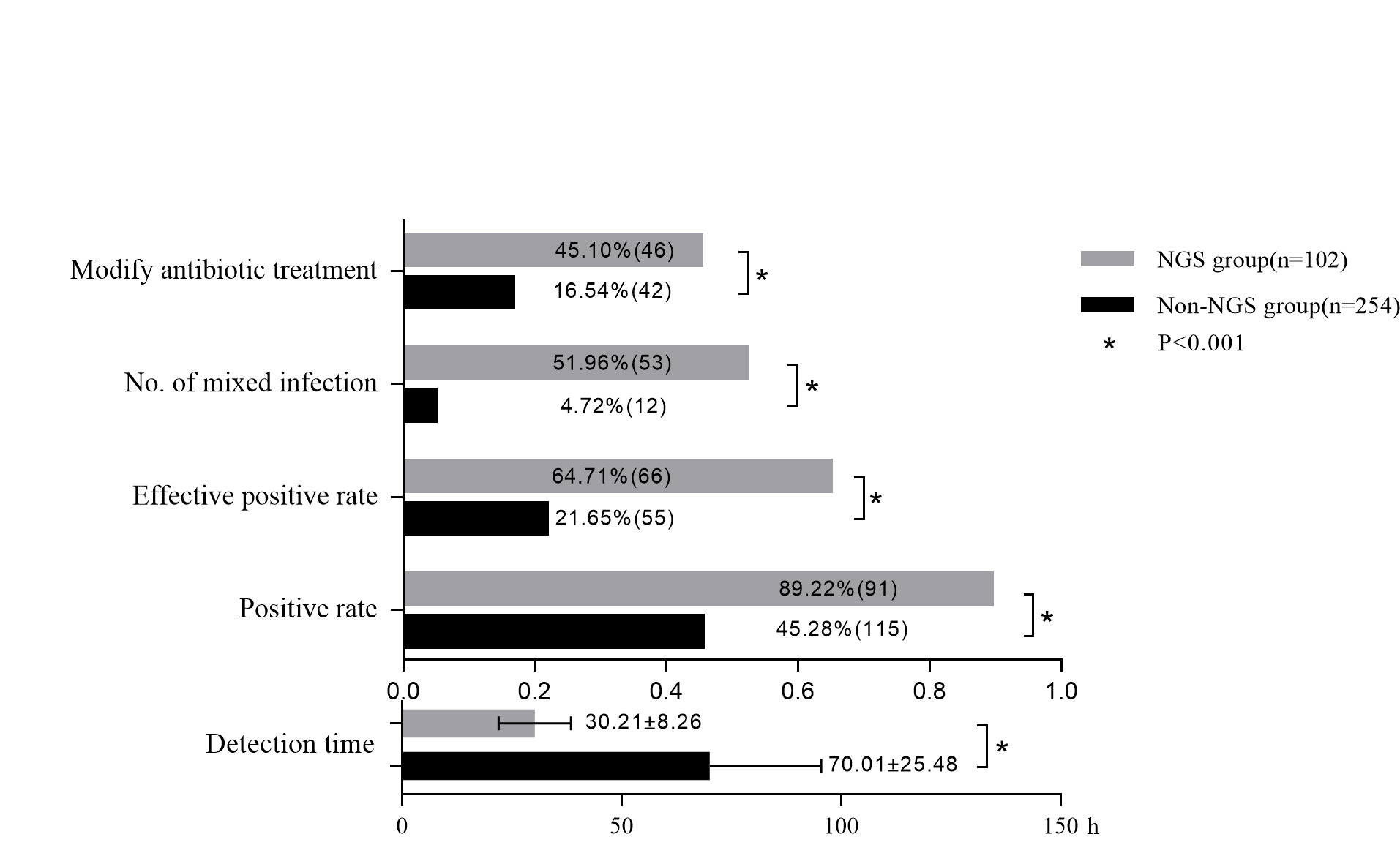

Supplement: Supplementary file 2 — Data S2. Supporting Information [file CRJ-16-793-s002.jpg]
