# Supplementary material for: The evaluation of next‐generation sequencing assisted pathogenic detection in immunocompromised hosts with pulmonary infection: A retrospective study
Source: Clin Respir J. 2022 Oct 18;16(12):793–801. doi: 10.1111/crj.13542 (PMC9716706; doi:10.1111/crj.13542)
Supplement: Supplementary file 3 — Data S3. Supporting Information [file CRJ-16-793-s004.pdf]

Supplementary material 3 Pathogens detected by NGS traditional microbial methods

a.

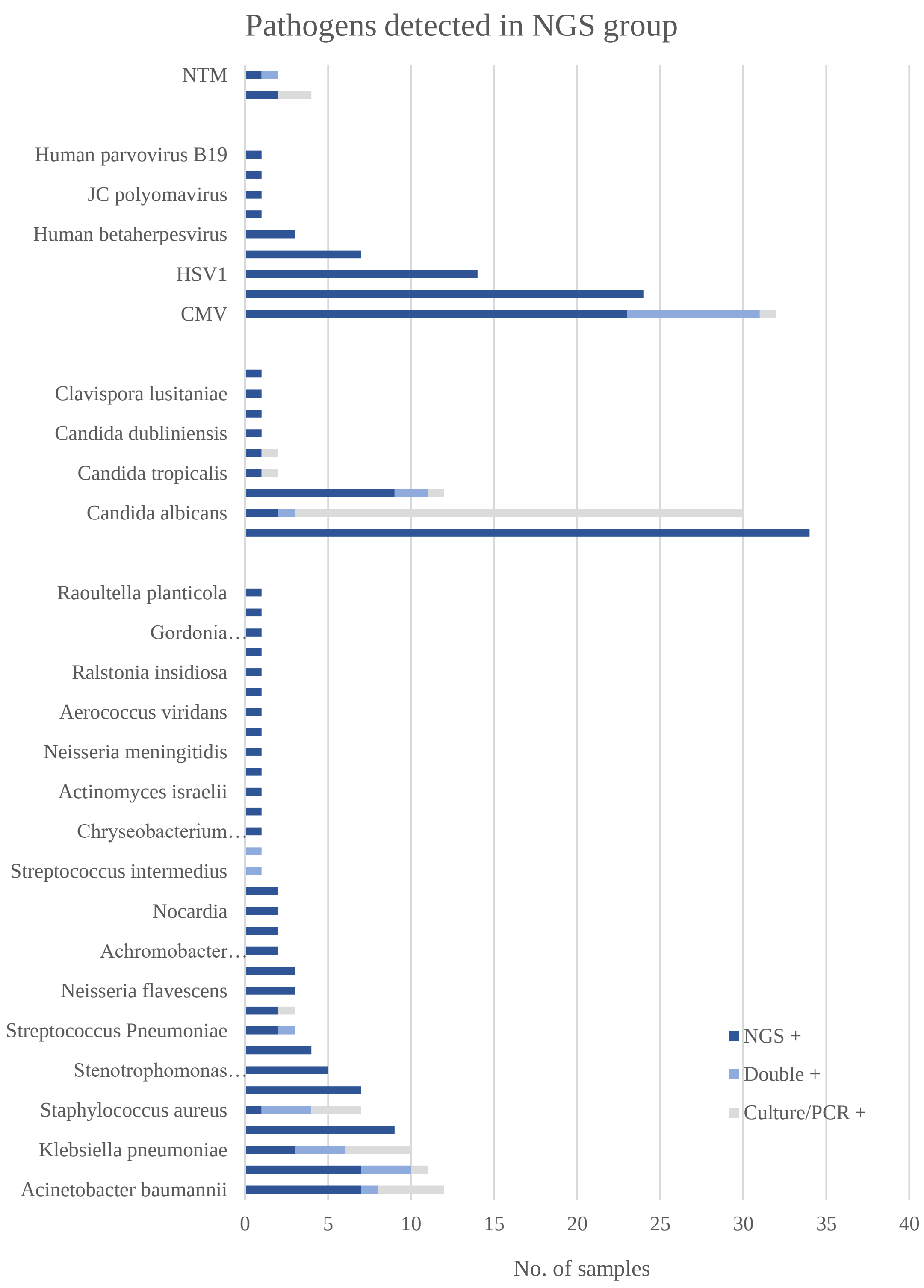

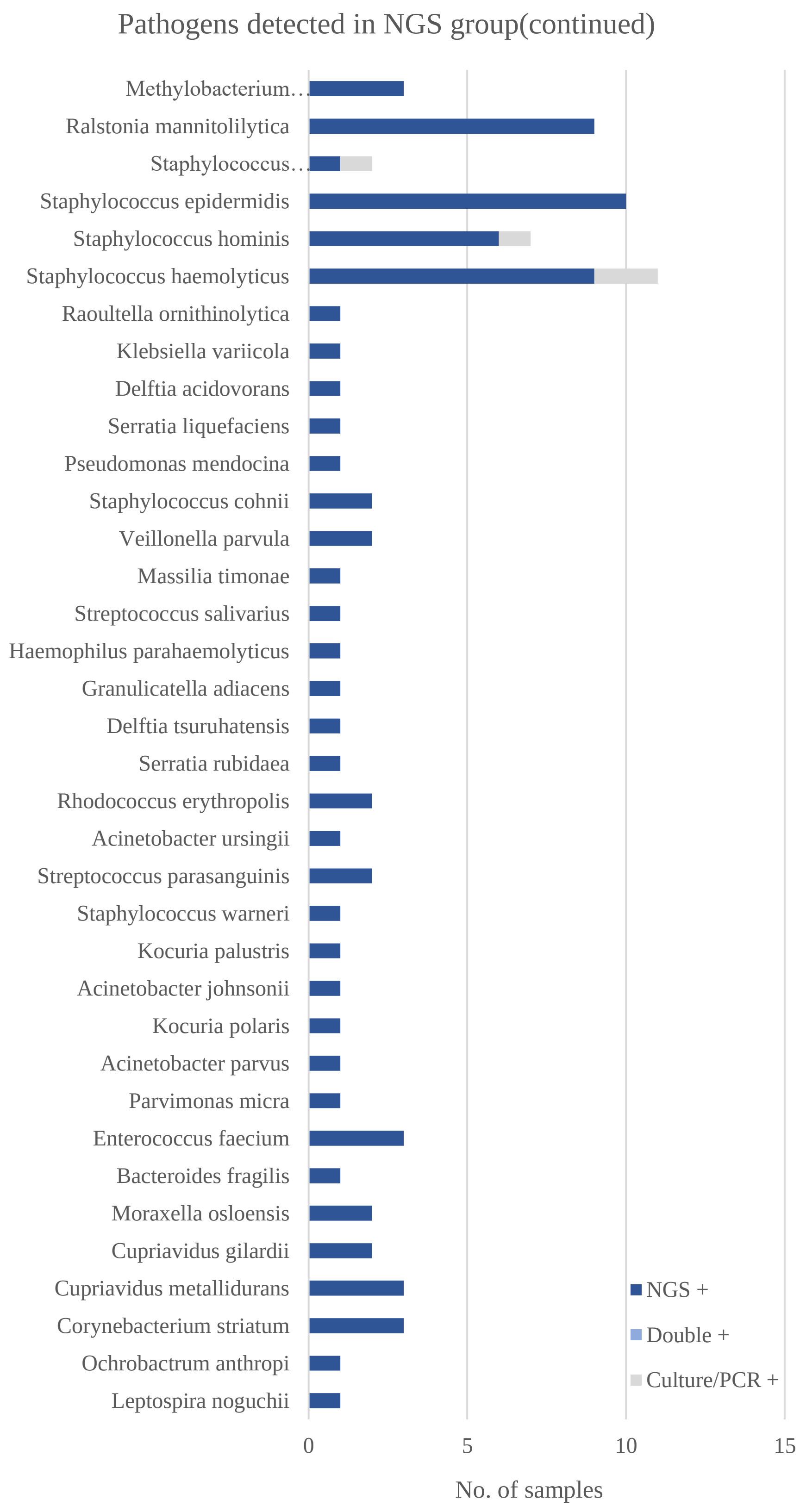

Pathogens detected in NGS group(continued)
Methylobacterium…
Ralstonia mannitolilytica
Staphylococcus…
Staphylococcus epidermidis
Staphylococcus hominis
Staphylococcus haemolyticus
Raoultella ornithinolytica
Klebsiella variicola
Delftia acidovorans
Serratia liquefaciens
Pseudomonas mendocina
Staphylococcus cohnii
Veillonella parvula
Massilia timonae
Streptococcus salivarius
Haemophilus parahaemolyticus
Granulicatella adiacens
Delftia tsuruhatensis
Serratia rubidaea
Rhodococcus erythropolis
Acinetobacter ursingii
Streptococcus parasanguinis
Staphylococcus warneri
Kocuria palustris
Acinetobacter johnsonii
Kocuria polaris
Acinetobacter parvus
Parvimonas micra
Enterococcus faecium
Bacteroides fragilis
Moraxella osloensis
Cupriavidus gilardii
Cupriavidus metallidurans
Corynebacterium striatum
Ochrobactrum anthropi
Leptospira noguchii
NGS +
Double +
Culture/PCR +
0
5
10
15
No. of samples

b.

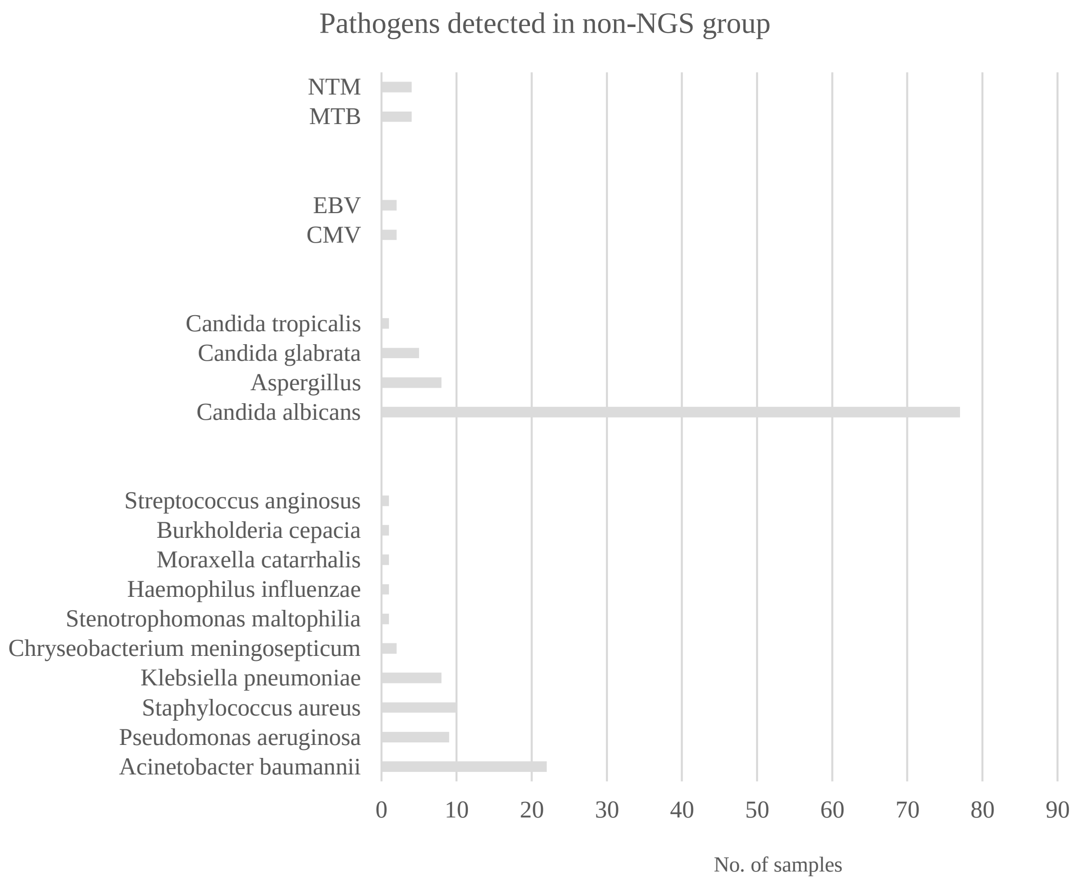

Pathogens detected in non-NGS group
NTM
MTB
EBV
CMV
Candida tropicalis
Candida glabrata
Aspergillus
Candida albicans
Streptococcus anginosus
Burkholderia cepacia
Moraxella catarrhalis
Haemophilus influenzae
Stenotrophomonas maltophilia
Chryseobacterium meningosepticum
Klebsiella pneumoniae
Staphylococcus aureus
Pseudomonas aeruginosa
Acinetobacter baumannii
0
10
20
30
40
50
60
70
80
90
No. of samples
